# A Hybrid Algorithm for Clustering of Time Series Data Based on Affinity Search Technique

**DOI:** 10.1155/2014/562194

**Published:** 2014-03-25

**Authors:** Saeed Aghabozorgi, Teh Ying Wah, Tutut Herawan, Hamid A. Jalab, Mohammad Amin Shaygan, Alireza Jalali

**Affiliations:** Faculty of Computer Science & Information Technology Building, University of Malaya, 50603 Kuala Lumpur, Malaysia

## Abstract

Time series clustering is an important solution to various problems in numerous fields of research, including business, medical science, and finance. However, conventional clustering algorithms are not practical for time series data because they are essentially designed for static data. This impracticality results in poor clustering accuracy in several systems. In this paper, a new hybrid clustering algorithm is proposed based on the similarity in shape of time series data. Time series data are first grouped as subclusters based on similarity in time. The subclusters are then merged using the *k*-Medoids algorithm based on similarity in shape. This model has two contributions: (1) it is more accurate than other conventional and hybrid approaches and (2) it determines the similarity in shape among time series data with a low complexity. To evaluate the accuracy of the proposed model, the model is tested extensively using syntactic and real-world time series datasets.

## 1. Introduction

Clustering is considered the most important unsupervised learning problem. The clustering of time series data is particularly advantageous in exploratory data analysis and summary generation. Time series clustering is also a preprocessing step in either another time series mining task or as part of a complex system. Researchers have shown that using well-known conventional algorithms in the clustering of static data, such as partitional and hierarchical clustering, generates clusters with an acceptable structural quality and consistency and is partially efficient in terms of execution time and accuracy [1]. However, classic machine learning and data mining algorithms are ineffective with regard to time series data because of the unique structure of time series, that is, its high dimensionality, very high feature correlation, and (typically) large amount of noise [2–4]. Accordingly, numerous research efforts have been conducted to present an efficient approach to time series clustering. However, the focus on the efficiency and scalability of these methods in handling time series data has come at the expense of losing the usability and effectiveness of clustering [5].

The clustering of time series data can be broadly classified into conventional approaches and hybrid approaches. Conventional approaches employed in the clustering of time series data are typically partitioning, hierarchical, or model-based algorithms. In hierarchical clustering, a nested hierarchy of similar objects is constructed based on a pairwise distance matrix [6]. Hierarchical clustering has great visualization power in time series clustering [7]. This characteristic has made hierarchical clustering very suitable for time series clustering [8, 9]. Additionally, hierarchical clustering does not require the number of clusters as an initial parameter, in contrast to most algorithms. This characteristic is a well-known and outstanding feature of this algorithm and is a strength point in time series clustering because defining the number of clusters is often difficult in real-world problems. However, hierarchical clustering is cumbersome when handling large time series datasets [10] because of its quadratic computational complexity. As a result of its poor scalability, hierarchical clustering is restricted to small datasets. On the other hand, partitioning algorithms, such as the well-known* k*-Means [11] or* k*-Medoids algorithm [12], are among the most used algorithms in this domain.* k*-Means and* k*-Medoids algorithms are very fast compared with hierarchical clustering [13], making them very suitable for time series clustering. Therefore, these algorithms have been used in several works, either in their “crispy” manner [3, 14–18] or in their “fuzzy” manner (Fuzzy* c*-Means and Fuzzy* c*-Medoids) [17–20]. Model-based clustering assumes a model for each cluster and determines the best data fit for that model. The model obtained from the generated data defines the clusters [21]. A few articles use model-based time series clustering [22–26]; however, two typical drawbacks have been discovered. First, the parameters should be set, and the parameter setting is based on the user's assumptions, which may be false and may result in inaccurate clusters. Second, model-based clustering has a slow processing time (especially neural networks) with respect to large datasets [27].

Aside from all of these conventional approaches, some new articles emphasize the enhancement of algorithms and present customized models (typically as a hybrid method) for time series data clustering. One of the latest works is an article by Lai et al. [28], who describe the problem of overlooked information as a result of dimension reduction. Lai et al. claim that the overlooked information can result in time series clustering results that have a different meaning. To solve this issue, they adopt a two-level clustering method, where both the whole time series and the subsequence of the time series are considered in the first and second levels, respectively. Lai et al. employed Symbolic Aggregate ApproXimation (SAX) [29] transformation as a dimension reduction method and the Cluster Affinity Search Technique (CAST) [30] as a first-level clustering algorithm to group first-level data. To measure distances between time series data in the second level, Dynamic Time Warping (DTW) [31] was used on data with varying lengths, and Euclidean distance (ED) was used on data of equal length. However, CAST algorithm is used twice in this approach, once to generate initial clusters and the other to split each cluster into subclusters, which is rather complex.

The authors in [32] also propose a new multilevel approach for shape-based time series clustering. First, time series data are selected from a generated one-nearest-neighbor network. To generate the time series network, the authors propose a triangle distance measurement to calculate the similarity between time series data. Hierarchical clustering is then performed on the selected time series data. Second, the data size is reduced by approximately 10% using this approach. This algorithm requires a nearest-neighbor network in the first level. The complexity in generating a nearest-neighbor network is *O*(*n* ^2^), which is rather high. As a result, the authors attempt to reduce the search area by data preclustering (using* k*-Means) and limit the search to each cluster only to reduce the creation network. However, generating the network itself remains costly, rendering it inapplicable in large datasets. Additionally, the solution to the challenge of generating the prototypes via* k*-Means when the triangle is used as a distance measure is unclear.

In this study, the low quality problem in existing works is addressed by the proposal of a new Two-step Time series Clustering (TTC) algorithm, which has a reasonable complexity. In the first step of the model, all the time series data are segmented into subclusters. Each subcluster is represented by a prototype generated based on the time series affinity factor. In the second step, the prototypes are combined to construct the ultimate clusters.

To evaluate the accuracy of the proposed model, TTC is tested extensively using published time series datasets from diverse domains. This model is shown to be more accurate than any of the existing works and overcomes the limitations of conventional clustering algorithms in determining the clusters of time series data that are similar in shape. With TTC, the clustering of time series data based on similarity in shape does not require calculation of the exact distances among all the time series data in a dataset; instead, accurate clusters can be obtained using prototypes of similar time series data.

The rest of this paper is organized as follows. In Section 2, some concepts and definitions are explained. In Section 3, the proposed model is described. In Section 4, the algorithm is applied on diverse time series datasets and the experimental results are analyzed. In Section 5, conclusions are drawn and future perspectives are discussed.

## 2. Concepts and Definitions 

The key terms used in this study are presented in this section. The objects in the dataset related to the problem at hand are time series data of similar lengths.

**Definition 1 (time series)**

A time series *F*_*i*_ = {*f*_1_,…, *f*_*t*_,…, *f*_*n*_} is an ordered set of numbers that indicate the temporal characteristics of objects at any time* t* of the total track life *T* [33].

**Definition 2 (time series clustering)**

Given a dataset of *N* objects, *D* = {*F*_1_, *F*_2_,…, *F*_*N*_}, where *F*_*i*_ is a time series. The unsupervised partitioning process of* D* into *C* = {*C*_1_, *C*_2_,…, *C*_*k*_} occurs such that homogenous time series data are grouped together based on similarity in shape, a grouping that is called time series clustering. *C*_*i*_ is then called a cluster, where *D* = ⋃_*i*=1_^*k*^ *C*_*i*_ and *C*_*i*_∩*C*_*j*_ = *∅*, for *i* ≠ *j*.

**Definition 3 (similarity in time)**

The similarity between two time series data is based on the similarity in each time step.

**Definition 4 (similarity in shape)**

The similarity between two time series is based on the similarities between their subsequences or their common trends regardless of time occurrence.

**Definition 5 (subcluster)**

A subcluster SC_*i*_ is a set of individual time series data that are similar in time and are represented as a single prototype. Time series data are attached to a new subcluster based on their affinity to the subcluster. Thus, *V* = {SC_1_, SC_2_,…, SC_*i*_,…, SC_*M*_} is the set of all subclusters, where *k* < *M* << *N*.

**Definition 6 (affinity)**

The affinity of a time series *F*_*x*_ with a subcluster SC_*i*_ is defined as follows:

(1) $$\begin{array}{c} a_{i}\left(F_{x}\right)=\frac{\sum_{y\in {\text{SC}}_{i}}^{}A_{xy}}{\left|{\text{SC}}_{i}\right|}, \end{array}$$

where *A*_*xy*_ is the similarity between time series *F*_*x*_ and *F*_*y*_ and |SC_*i*_| is the number of time series data that exist in the subcluster SC_*i*_. This value is used to distinguish the time series data that have a low affinity by placing them into a new subcluster.

**Definition 7 (prototype)**

The prototype is a time series *R*_*i*_ = {*r*_1_,…, *r*_*x*_,…, *r*_*n*_}, which represents the most typical time point of a finite set of time series data in subcluster SC_*i*_. The prototype of each subcluster is constructed with regard to the affinity of each time series with the subcluster.

Time series clustering relies highly on a distance measure. Several distance measures have been proposed by researchers in the literature [34–42]. However, ED and DTW are revealed to be the most common methods used in time series clustering because of the efficiency of ED and the effectiveness of DTW in similarity measurement. Simple and fast, ED is used as benchmark in numerous studies (approximately 80%) [34, 43–45] because it is parameter-free. However, it is not the best choice as a distance function because it is extremely dependent on the domain of the problem at hand and the dataset's time series characteristics. In fact, ED is very weak and sensitive to slight shifts across the time axis [46–49], which limits it in terms of determining time series data that are* similar in time*.

**Figure 1:**
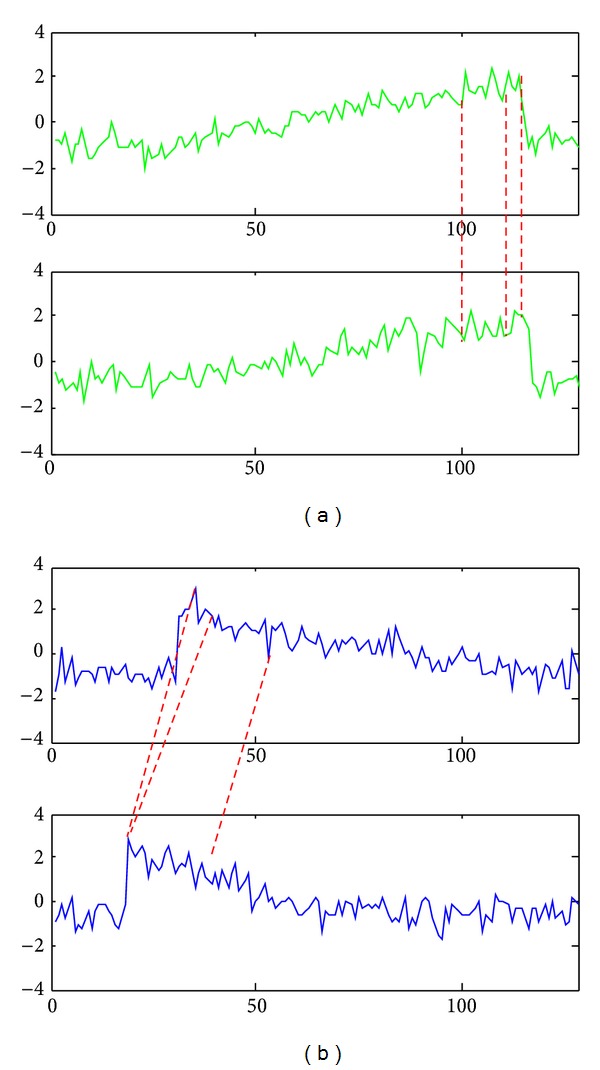
Similarity in shape (b) and similarity in time (a) between two time series data.

In contrast to ED, which proposes one-to-one matching between time points, DTW is suggested as a one-to-many measurement. DTW is a generalization of ED, which solves the local shift problem in the time series data to be compared (see Figure 1). The local shift problem is a time scale issue that characterizes most time series data. Handling local shifts allows similar shapes to be matched even if they are out of phase in the time axis; that is, they are* similar in shape*.

Using this definition, time series clusters with similar patterns of change are constructed regardless of time points, for example, to cluster share prices related to different companies that have a common stock pattern independent of time series occurrence [22, 50]. DTW is thus superior to ED [31, 39, 41, 51, 52], as the latter can only determine time series that are similar in time.

DTW “warps” the time axis to achieve the best alignment between data points within the series. Dynamic programming is generally used to effectively determine the warping path. However, warping causes a scalability problem that requires quadratic computation, which is a huge challenge for DTW [53]. However, we do not need to calculate all of the distances when the proposed algorithm previously mentioned is used; therefore, DTW can be adopted without affecting clustering efficiency.

## 3. The Proposed Algorithm

**Figure 2:**
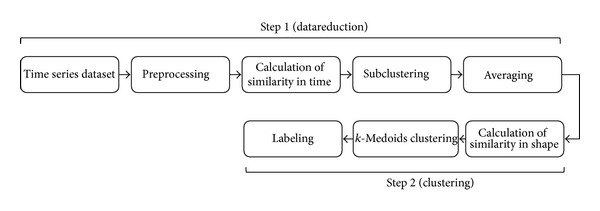
Block diagram for the proposed TTC algorithm.

The detailed description of the proposed algorithm is presented in this section.  Figure 2 shows the block diagram for the proposed TTC algorithm. First, the size of the time series dataset is reduced (i.e., data reduction) using the concept of affinity. A prototype is then generated for each subcluster. Consequently, subclusters are merged using* k*-Medoids clustering.

According to the steps above, the activities of the TTC are explained in the following sections.

### 3.1. Step 1: Data Reduction

The main objective of this TTC step is to reduce the size of the dataset by defining a prototype for each group of very similar time series data, which significantly decreases the complexity of TTC. The time series data are first standardized using* z*-score (*z*-normalization) [54], which causes the time series data to be invariant to scale and offset. Supposing that *F*_*i*_ = {*f*_1_, .., *f*_*t*_, .., *f*_*n*_} is a time series with *T* data points,* z*-normalization is defined as

(2) $$\begin{array}{c} z\text{-Normalization}\left(F_{i},\mu_{i},\mathrm{sd}\right)=\frac{f_{t}-\mu_{i}}{\mathrm{sd}}, \end{array}$$

where

(3) $$\begin{array}{c} \mu_{i}=\frac{\sum_{t=1}^{n}f_{t}}{n},\\ \mathrm{sd}=\sqrt{\frac{{\sum_{t=1}^{n}f_{t}\left(f_{t}-\mu_{i}\right)}^{2}}{n}}, \end{array}$$

where *μ*_*i*_ is an arithmetic mean of data points *f*_1_ through *f*_*n*_ and sd is the standard deviation of all the data points in the given time series.

**Figure 3:**
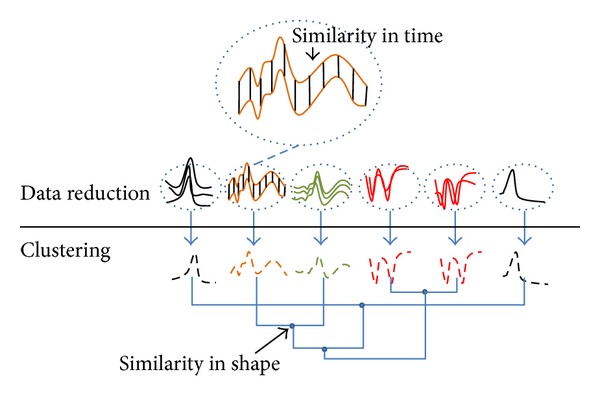
Reasoning behind the use of DTW in calculating similarity in shape between the prototypes of subclusters in the second step of TTC.

Subsequently, all the data are clustered as a whole based on similarity in time. In this step, the affinity search technique concept in CAST [30] is borrowed to generate the subclusters. CAST was essentially introduced into the bioinformatics domain for gene expression clustering; it is used in this step because the number of clusters does not need to be predetermined in CAST. In contrast to numerous algorithms that require the number of clusters to be predefined in advance, the mechanism used by the CAST algorithm can determine clusters dynamically and deal effectively with outliers. CAST works based on the pairwise similarity matrix of objects. The similarities between time series data are calculated and stored in an* N*-by-*N* similarity matrix (*A*_*N*×*N*_), where *A*_*ij*_ is the similarity between time series *F*_*i*_ and time series *F*_*j*_. ED is used as the dissimilarity measure to calculate the similarity (similarity in time) between time series data.  Figure 3 illustrates the reasoning behind the use of ED to construct subclusters in the first step. *A*_*N*×*N*_′ is assumed to be the pairwise distance matrix, where *A*_*ij*_′ is the Euclidian distance between *F*_*i*_ and *F*_*j*_. This distance is mathematically defined as

(4) $$\begin{array}{c} {A_{ij}^{\prime }=\text{dis}}_{\text{ED}}\left(F_{i},F_{j}\right)=\sqrt{\overset{n}{\underset{i=1}{\sum }}{\left(f_{i}-f_{j}\right)}^{2}}, \end{array}$$

where the square root step can be removed because the square root function is monotonic and reverts to the same rankings in clustering [2]. The time complexity of this calculation can also be reduced from linear to constant by caching some of the calculated results [55]. Given *A*_*N*×*N*_, the algorithm is performed by adding and removing time series data from a subcluster based on a threshold affinity value between 0 and 1, as defined by the user.

A new subcluster (Definition 5) is constructed by the time series datum that has the highest similarity to other time series data. Subsequently, each time series datum is added to a new subcluster based on its affinity with the subcluster (Definition 6); that is, each subcluster is constructed with a time series datum and is gradually completed by the addition of new time series data to the subcluster based on the average similarity (affinity) between the unassigned time series data and the current subcluster members. As previously mentioned, subclusters are formed sequentially with an affinity threshold. By defining the specific threshold value, the cluster accepts the high affinity time series datum. The affinity threshold *α* is specified to determine what is considered significantly similar. This parameter controls the number and sizes of the produced subclusters. After a subcluster is formed, CAST deletes the low affinity objects from the subcluster. The process of adding to and removing from a subcluster is performed consecutively until no further changes occur in the subcluster.

After each subcluster is constructed, a prototype is defined for each subcluster. The construction of an effective time series prototype is a vexing problem [56, 57]. In the current study, we propose a novel approach to represent time series data in a cluster. The prototype of each subcluster is calculated based on the affinity of each time series datum with the subcluster. An affinity set is maintained during the subclustering process for all the time series data, denoted as *a*_*i*_ (Definition 6). The affinity of a time series datum evidently implies its weight in the construction of the prototype. Given the subcluster SC_*i*_, its prototype is defined by a time series *R*_*i*_ = {*r*_1_,…, *r*_*x*_,…, *r*_*n*_}. *r*_*x*_ is then calculated as

(5) $$\begin{array}{c} r_{x}=\frac{\sum_{y\in {\text{SC}}_{i}}^{}a_{i}\left(F_{y}\right)*f_{yx}}{\left|{\text{SC}}_{i}\right|}, \end{array}$$

where *F*_*y*_ = {*f*_*y*1_,…, *f*_*yx*_,…, *f*_*yn*_} is a time series datum in CS_*i*_ and |SC_*i*_ | indicates the number of time series data in the subcluster.

### 3.2. Step 2: Clustering

In the first step, the time series data are grouped based on the similarity in time. However, two time series data that are not similar in time may be similar in shape. Similarity in shape is desirable in time series clustering because the constructed clusters are very close to the ground truth and are more meaningful. However, the methods that have this feature, such as DTW, are often costly [53] in the similarity evaluation of time series data. As a result, several researchers, such as [47, 58–61], try to accelerate the process, typically by proposing efficient lower bound approximations of DTW distance to reduce its complexity. However, most of these works are under the classification problem (the search area is pruned using a lower bound distance of DTW) and are not suitable for several clustering algorithms, where the dissimilarity matrix must be fully calculated. For example, in clustering algorithms such as* k*-Medoids or Unweighted Pair-Group Method with Arithmetic Mean [62], all distances must be calculated and no pruning can be performed. In such cases, the clustering process benefits from a fast and accurate similarity measure [63]. However, we do not have to calculate similarity in shape between all time series data in the TTC because the very close time series data (similar in time) are similar in shape as well. That is, the dissimilarity matrix does not need to be fully calculated using an expensive similarity measure such as DTW. As a result, only a small part of the matrix is calculated by DTW using the prototypes of the subclusters, which are small in size, in the first step of TTC (instead of all the data as a whole).  Figure 3 depicts the reasoning behind the use of ED and DTW in the first and second steps of TTC, respectively. As this figure shows, the intersimilarity between the time series data in the subclusters is computed based on similarity in time, and intrasimilarity is calculated based on similarity in shape.

**Pseudocode 1:**
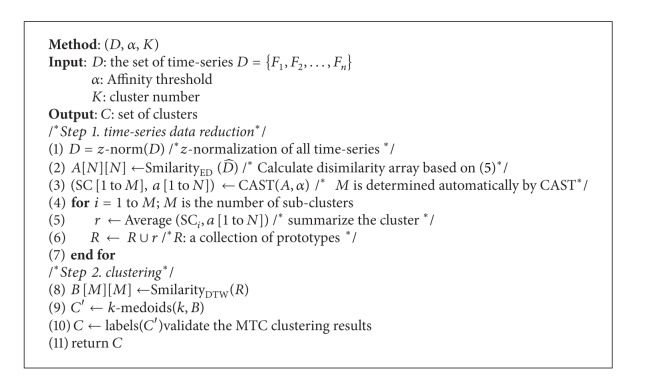
Pseudocode related to TTC.

Therefore, the similarity between subclusters is calculated and stored in an* M*-by-*M* similarity matrix *B*_*M*×*M*_, where *B*_*ij*_ is the similarity between the prototypes of subclusters SC_*i*_ and SC_*j*_. First, DTW distance among the prototypes of the subclusters is calculated to construct the pairwise dissimilarity matrix *B*_*M*×*M*_, where *B*_*ij*_ is the DTW distance of two subclusters' prototypes, namely, prototype *R*_*i*_ and prototype *R*_*j*_, as denoted by dis_DTW_. Suppose that *R*_*x*_ = {*r*_*x*1_,…, *r*_*xi*_,…, *r*_*xn*_} is the prototype of SC_*x*_, where* n* is the length of the prototype and *r*_*x*_ is calculated by (5). To compute the distance between the prototypes of SC_*x*_ and SC_*y*_, an *n* × *n* matrix is constructed for the distance of all pairs as* Z*(*R*_*x*_, *R*_*y*_), where *Z*_*i*,*j*_ = dis_ED_(*r*_*xi*_, *r*_*yj*_) and dis_ED_( ) is the Euclidean distance. Given *W* = {*w*_1_, *w*_2_, …, *w*_*u*_} as a set of warping paths, where *w*_*u*_ = {(*r*_*x*1_, *r*_*y*1_), (*r*_*xi*_, *r*_*yj*_),…, (*r*_*xn*_, *r*_*yn*_)} is a set of points that define a traversal of matrix* Z* and the DTW between the two prototypes *R*_*x*_ and *R*_*y*_ is a warping path that minimizes the distance between *R*_*x*_ and *R*_*y*_,

(6) $$\begin{array}{c} {\text{dis}}_{\text{DTW}}\left(R_{x},R_{y}\right)=\min\left(\overset{U}{\underset{u=1}{\sum }}\frac{W_{u}}{U}\right), \end{array}$$

where (*r*_*x*1_, *r*_*y*1_) = (1, 1) and (*r*_*xn*_, *r*_*yn*_) = (*n*,* n*) and 0 ≤ *r*_*xi*+1_ − *r*_*xi*_ ≤ 1 and 0 ≤ *r*_*yj*_ − *r*_*yj*+1_ ≤ 1, for all *i* < *n*.

Given the pairwise dissimilarity matrix, different schemes can be used for clustering.* k*-Medoids, which has been shown to be effective in the time series clustering domain [29–33], is selected. The TTC algorithm is presented in Pseudocode 1.

## 4. Analysis

### 4.1. Evaluation Metrics and Experimental Setup

The experiment on the proposed model is conducted with one syntactic dataset and 12 real-word datasets obtained from the UCR Time Series Data Mining Archive in various domains and sizes [64]. This set is selected because it is composed of various numbers of clusters with different cluster shapes and density, contains noise points, and is used as a benchmark in several articles in previous literature.

**Figure 4:**
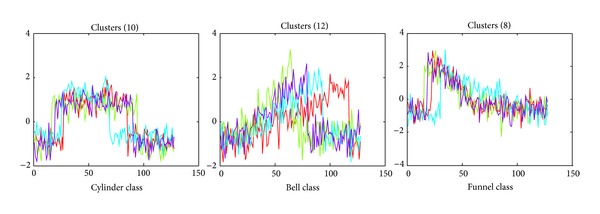
Three samples of each Cylinder, Bell, and Funnel (CBF) class dataset.

The well-known three-class Cylinder-Bell-Funnel (CBF) dataset is used as a syntactic dataset in the experiment on 2PTC with large datasets. The CBF dataset is an artificial dataset that has temporal domain properties and was originally proposed by Saito in 2000. This dataset has been used in numerous works [32, 65, 66]. It includes three types of time series data: Cylinder (c), Bell (b), and Funnel (f). Different CBF datasets are generated and used in this study. Examples of CBF time series datasets are shown in Figure 4.

In general, evaluating extracted clusters (patterns) is not easy in the absence of data labels [66]. However, all the selected datasets in this study have class labels (ground truth) and can be applied in evaluating TTC using external indices. The most commonly used external indices in the time series clustering domain are used in evaluating the accuracy of TTC, namely, Rand Index and Entropy. (The interested reader may refer to [67, 68] for definitions.)

Rand Index is a popular quality measure [69–71] for evaluating time series clusters; it measures the agreement between two partitions, that is, how close clustering results are to the ground truth. The agreement between cluster *C* and ground truth *G* can be estimated using

(7) $$\begin{array}{c} \text{RI}\left(C,G\right)=\sqrt{\frac{\left|\text{TP}\right|+\left|\text{TN}\right|}{\left|\text{TP}\right|+\left|\text{TN}\right|+\left|\text{FP}\right|+\left|\text{FN}\right|}}, \end{array}$$

where |TP| (True Positive) is the number of pairs belonging to one class in *G* (ground truth) and are clustered together in *C*. |TN| (True Negative) is the number of pairs that neither belong to the same class in *G* nor are clustered together in *C*. The types of error clustering are the |FN| (False Negative), which is the number of pairs that belong to one class in *G* but are not clustered together in *C*, and |FP| (False Positive), which is the number of pairs that do not belong to one class in *G* (dissimilar time series) but are clustered together in *C*. The Random Index evaluation criteria have values ranging from 0 to 1, where 1 corresponds to the case wherein ground truth and clustering result are identical and 0 corresponds to the case wherein they are completely different.

The Entropy [72, 73] of a cluster shows the dispersion of classes within a cluster (this dispersion should be low) in several domains. Entropy has been adopted in the evaluation of time series clustering in literature [74, 75] as well. It is a function of the distribution of classes in the resulting clusters. For each cluster *C*_*j*_, the class distribution of data is computed as the probability Pr(*G*_*i*_ | *C*_*j*_), wherein an instance in *C*_*j*_ belongs to class *G*_*i*_. Using this class distribution, the normalized entropy of *C*_*j*_ is computed as

(8) $$\begin{array}{c} \text{Entropy}\left(C_{j}\right)=-\frac{1}{\log h}\overset{h}{\underset{i=1}{\sum }}\text{Pr}\left(\left(G_{i}\;|\;G_{j}\right)\times \log\left(G_{i}\;|\;G_{j}\right)\right), \end{array}$$

where Pr(*G*_*i*_ | *C*_*j*_) = |*C*_*j*_∩*G*_*i*_|/|*C*_*j*_|. ConEntropy is the converse of Entropy based on the definition of Entropy, wherein ConEntropy is 1 when the ground truth and the clustering result are identical. Overall ConEntropy (*E* ∈ [0, 1]) is defined as the sum of the individual cluster entropies weighted by the size of each cluster:

(9) $$\begin{array}{c} \text{ConEntropy}\left(C,G\right)=1-\frac{1}{\left|D\right|}\overset{K}{\underset{j=1}{\sum }}\left|C_{j}\right|\times \text{Entropy}\left(C_{j}\right). \end{array}$$

Based on the measures above, a good clustering solution is expected to have high ConEntropy. To avoid a biased evaluation, the conclusions are drawn based on the average value of the indices. Although the focus of this study is improving the accuracy of TTC, the scalability of the proposed model is also calculated to prove its theoretical feasibility.

### 4.2. Accuracy Evaluation

In this section, the results are compared with those of partitional clustering. First, the distance between the time series data is calculated using ED to compare TTC with conventional* k*-Medoids. The reader may wonder why DTW is not used to compare the results. In simple terms, the use of DTW does not result in a fair comparison because DTW is not practically feasible in the real world as a result of its very high complexity. The complexity of DTW in between each pair of time series data in* k*-Medoids is *O*(*Ik*(*N* − *k*)^2^ and *O*(*n* ^2^), where *N* is the number of time series data, *k* is the number of clusters,* I* is the number of iterations required for convergence, and *n* is the length of time series data. Therefore, the total computation of* k*-Medoids is *O*(*Ik*(*N*−*k*)^2^ · *n* ^2^). That is, *N*(*N* − 1)/2 distance calculation is required to calculate the confusion matrix alone (needed in clustering), where* N* is the number of time series. As a result, the complexity of the distance matrix alone (not the entire clustering process) equals *N*(*N* − 1)*n* ^2^/2, which is very high. For example, given* N* = 1000 and* n* = 152 in a dataset, the number of instruction executions is 11,540,448,000. However, using TTC on the same process requires approximately 177,084,440 executions because the process operates on a fraction of the entire dataset with the reduction factor = 0.1 (see (11)).

As a fair comparison in the subsequent experiment, the raw time series data are represented by a representation method because time series data are represented by a representation method prior to clustering in the majority of previous literature. Numerous studies focusing on the representation or dimensionality reduction of time series data have been conducted [7, 55, 59]. Among these representation methods, each of which has its strong points and weaknesses, Piecewise Aggregate Approximation (PAA) [52, 75] is adopted in this study because of its strength in the representation of time series data and its low complexity [76]. The raw time series data are represented using different compression ratios (compression_ratio_ = [4,6, 8]) because the accuracy of PAA itself depends on the number of segmentations. As a result, the mean of three accuracies for each dataset is calculated as the average accuracy of* k*-Medoids.  Table 1 shows the quality of the TTC approach against quality of the* k*-Medoids with regard to raw time series data and the time series data represented by PAA.

As expected, the comparison of TTC with* k-Medoids (ED)* and* k-Medoids (PAA-ED)* shows that TTC is more accurate in most of the datasets. TTC outperforms* k-Medoids (ED)* because ED cannot handle the local shifts in time series data, which decreases the accuracy of the final clusters.

Furthermore, TTC is more accurate than the conventional* k*-Medoids on represented time series, that is,* k-Medoids (PAA-ED)*. Although several outliers and noises in raw time series data are handled in the time series represented by PAA, the proposed algorithm, namely, TTC, remains superior to* k-Medoids (PAA-ED) *because of its shift-handling mechanism. The result shows that improved clustering quality is obtainable without reducing the time series dimension by using the prototypes of very similar time series data. This result is the proof of the researcher's claim that the TTC model can outperform conventional algorithms using either raw time series data or dimensionality reduction approaches.

### 4.3. Comparing TTC with Hybrid Models

**Figure 5:**
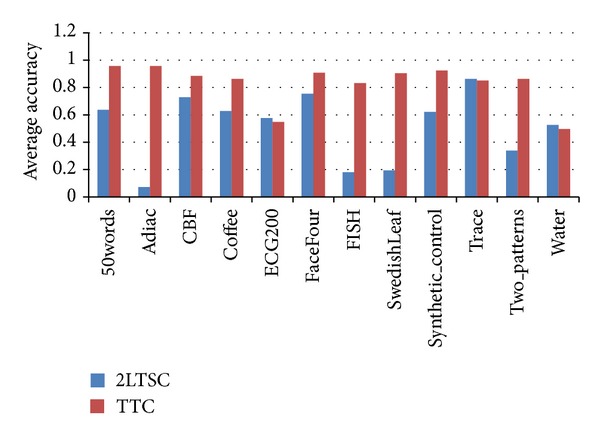
Comparison of 2LTSC and TTC against ground truth using the test datasets.

As mentioned in related works, one of the novel works close to the proposed model in this study is the two-level approach proposed by Lai et al. [28] called the 2LTSC. In Lai et al.'s work, SAX transformation is used as a dimension reduction method, and CAST is used as the clustering algorithm in the first level. In the second level, DTW is used to calculate distances between time series data with varying lengths, and ED is used to calculate distances between data of equal length. The 2LTSC works with the CAST algorithm, wherein the number of clusters is indirectly determined by a threshold. Hence, the same number of clusters (generated by 2LTSC) is also used in TTC after the 2LTSC is run.  Figure 5 shows the best clustering result of both approaches.

As mentioned, a high-resolution time series is used in the TTC model, which is superior to the dimensionality reduced time series used in 2LTSC. As a result, the quality of TTC is increased after clustering occurs in the second level. The subclusters are merged in the second step of TTC, which causes the generated cluster structure to be more similar to the ground truth.

**Figure 6:**
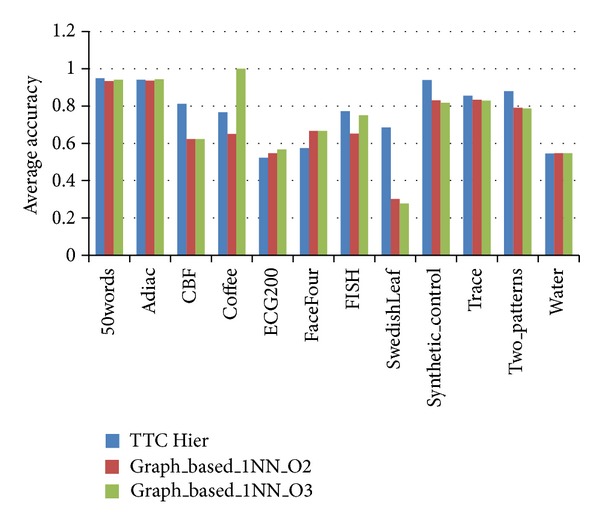
Quality of clustering using the graph-based approach as compared with TTC.

Another study that performed clustering in more than one step is Zhang et al. [32], which was discussed in the literature review. As previously mentioned, Zhang et al. proposed a new multilevel approach for shape-based time series clustering, wherein several candidate time series data are selected and clustered. To compare this approach (called the graph-based approach) with the TTC model, the quality of TTC clustering in terms of different orders of the nearest-neighbor network is calculated and shown in Figure 6. To provide fair conditions, the order of two to three is considered in the graph-based approach, which provides a reasonable reduction in the second layer.

As the result shows, TTC is superior to the graph-based algorithm in some datasets. The graph-based approach notably requires the generation of a nearest-neighbor graph, which is costly. However, the graph-based approach can be advantageous in datasets where similarity in time is essentially very important, such as the Coffee dataset (as shown in Figure 6). To summarize, the proposed model, namely, TTC, can outperform rival approaches, even with lower time complexity.

### 4.4. Data Reduction

To verify the effect of data reduction on final clustering, some experiments are conducted. In this experiment, we calculate the error rate in the data reduction step based on the CAST parameter, that is, the affinity threshold. Different sizes of the syntactic dataset CBF are used in this experiment.

First, a parameter is defined as reduction factor *R*_factor_

(10) $$\begin{array}{c} R_{\text{factor}}=\frac{M}{N}, \end{array}$$

where *N* is the size of the dataset and *M* is the number of subclusters generated by CAST (referred to as the number of prototypes).

The error rate *E*_rate_ of the subclusters is calculated based on the number of items in the same subcluster that belongs to the same class (ground truth) [77]. Given *G* = {*G*_1_, *G*_2_,…, *G*_*M*_} as ground truth clusters and *V* = {SC_1_, SC_2_,…, SC_*i*_,…, SC_*M*_} as the subclusters generated by CAST, the subcluster SC_*i*_ is assigned to the class most frequently found in the cluster to compute the error rate of cluster SC_*i*_ with respect to *G*. The error rate of this assignment is then measured by counting the number of misclassified time series data and dividing the result by the number of time series data in the subcluster.* M* is assumed to be the number of subclusters determined by CAST, and the size of cluster *C*_*i*_ is shown by |SC_*i*_|. max⁡⁡(|SC_*i*_∩*G*_*j*_|) is assumed to denote the number of items in subcluster SC_*i*_ that are not in *G*_*j*_. The error rate of cluster SC_*i*_ is then given by

(11) $$\begin{array}{c} E_{\text{rate}}\left({\text{SC}}_{i}\right)=\frac{1}{\left|\text{S}{\text{C}}_{i}\right|}\max\left(\left|{\text{SC}}_{i}\cap G_{j}\right|\right). \end{array}$$

Given *N* as the size of the dataset, the overall error rate of the reduction step can be expressed as a weighted sum of individual cluster error rates:

(12) $$\begin{array}{c} E_{\text{rate}}=\overset{M}{\underset{i=1}{\sum }}\frac{\left|{\text{SC}}_{i}\right|}{N}E_{r}\left({\text{SC}}_{i}\right). \end{array}$$

**Figure 7:**
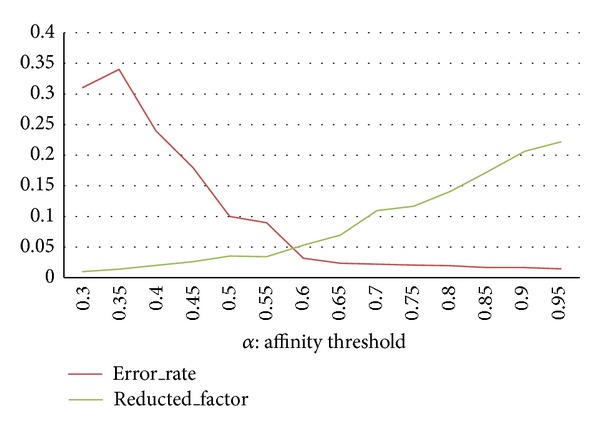
Reduction factor and error rate of the TTC approach across affinity threshold values for the CBF dataset.

As previously mentioned, the affinity threshold in the TTC algorithm determines the size and shape of sub clusters. If the value of the threshold is high, sub clusters are denser and the number of prototypes increases. As a result, the reduction factor increases.  Figure 7 shows the reduction factor and error rate of TTC across different affinity threshold values. The result shows that a good trade-off between reduction factor and error rate is obtained in thresholds above 0.7 for both datasets. As the threshold value increases, a lower error rate is encountered. The number of subclusters also increases (and reduction factor is higher). The following experiment verifies that TTC can reduce the data size by approximately 77% (*R*_factor_ = 0.23). The effectiveness of TTC is not significantly reduced; that is, the error rate is less than 0.05.

### 4.5. Evaluation of TTC on Large Datasets

**Figure 8:**
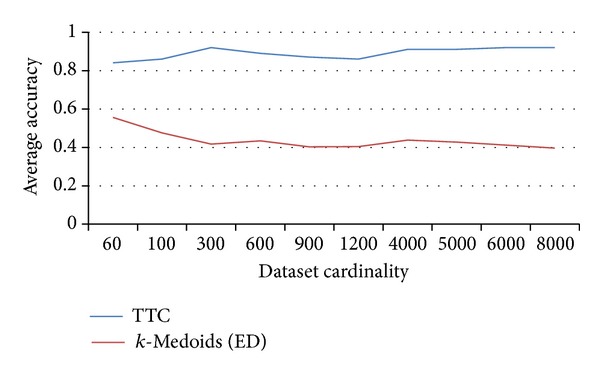
Accuracy of TTC across different CBF dataset sizes.

**Figure 9:**
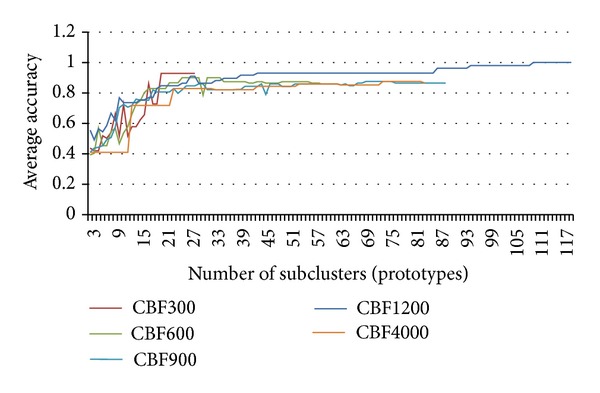
Accuracy of TTC across different numbers of subclusters.

To confirm the effectiveness of TTC further, some experiments are conducted on large synthetic datasets. For this purpose, up to 8,000 CBF time series are generated. To evaluate the results of the proposed model on large datasets, the average accuracy of TTC with regard to different CBF data sizes is shown in Figure 8. The experiment on TTC was also conducted with respect to different numbers of subclusters. This experiment shows the accuracy of TTC on large datasets. The average accuracy of TTC with respect to different numbers of subclusters is shown in Figures 8 and 9.

As the result shows, the quality of TTC is superior to that of other algorithms. The quality of TTC reaches 90% (Figure 9) in most of the cardinalities of the dataset when 30 subclusters are used. The maximum accuracy of conventional approaches is approximately 50% (Figure 8). The trend shows an increase in quality as the size of the dataset increases (Figure 8). Therefore, the use of DTW is not necessary in the clustering of all the data in very large datasets; it can be applied to smaller sets of a time series subset represented by prototyping instead.

## 5. Conclusion and Future Works

We illustrated the advantages of using some time series data as prototypes to cluster time series data based on the similarity in shape. We proposed a two-step clustering approach and showed its usage. The results obtained by applying TTC to different datasets were evaluated extensively. Clustering can be applied to a large time series dataset to generate accurate clusters. In the experiments with various datasets, different evaluation methods were used to show that TTC outperforms other conventional and hybrid clustering. Currently, we are working on a multistep approach, which is very scalable in the clustering of very large time series datasets. This approach will be performed as an anytime split-and-merge algorithm to present early results to the user and thus improve the clusters.

## Figures and Tables

**Table 1:** Quality of TTC approach against the standard *k*-Medoids with regard to raw time series data and the time series data represented by PAA.

Dataset	Number of classes	DS size	Length	*k*-Medoids (ED)		*k*-Medoids (PAA-ED)		TTC	
				RI	ConEnrtropy	RI	ConEnrtropy	RI	ConEnrtropy
50words	50	455	270	0.95	0.73	0.95	0.73	0.96	0.79
Adiac	37	391	176	0.94	0.58	0.94	0.58	0.96	0.64
CBF	3	900	128	0.67	0.29	0.7	0.41	0.88	0.78
Coffee	2	28	286	0.8	0.6	0.78	0.54	0.86	0.68
ECG200	2	100	96	0.61	0.18	0.61	0.18	0.55	0.14
FaceFour	4	88	350	0.77	0.51	0.77	0.51	0.91	0.78
FISH	7	175	463	0.77	0.32	0.8	0.32	0.83	0.48
SwedishLeaf	15	625	128	0.9	0.54	0.9	0.54	0.9	0.58
synthetic_control	6	300	60	0.82	0.56	0.87	0.79	0.92	0.95
Trace	4	100	275	0.75	0.51	0.78	0.59	0.85	0.78
Two_Patterns	4	4000	128	0.63	0.03	0.69	0.32	0.86	0.88
Wafer	2	6164	152	0.43	0.01	0.43	0.01	0.5	0.21
